# Supplementary material for: Development of a fixed module repertoire for the analysis and interpretation of blood transcriptome data
Source: Nat Commun. 2021 Jul 19;12:4385. doi: 10.1038/s41467-021-24584-w (PMC8289976; doi:10.1038/s41467-021-24584-w)
Supplement: Supplementary file 1 — Supplementary Information [file 41467_2021_24584_MOESM1_ESM.pdf]

<u>Supplementary Methods</u>: Module generation and pseudocode

Each of the preprocessed microarray datasets was clustered in parallel using Euclidean distance and the Hartigan's K-Means clustering algorithm, a hybrid of hierarchical and k-means clustering algorithms. An additional modification was made to the algorithm. If at any k the algorithm creates a cluster whose members' average Pearson correlation to the mean cluster vector is <0.3, the cluster is deleted and the algorithm begins again at k-1. The 'ideal' number of clusters (k) for each dataset was determined within a range of k=1-100 by means of the jump statistic.

Taking the 16 sets of clusters as the input, a weighted co-cluster graph — a probe by probe matrix where the value of each cell is set to a 16-bit bitmask (each bit corresponding to a dataset) indicating in which datasets the probes were co-clustered — was created. The bit was set to 1 if the probe pair co-clusters in the corresponding dataset, and to 0 if not. Therefore, the number of times the probes co-clustered (the weight) is equivalent to the number of bits in the mask that are set to 1. At this point, the goal is to extract sets of probes that are most frequently clustered together in the same datasets, proceeding from the most stringent requirements to the least. To accomplish this, an iterative algorithm was used. To begin, the maximum clique threshold was initialized to the number of input cluster sets, the paraclique threshold (pt) was calculated, and a minimum seed size was chosen (we used fifteen). The outer loop began by creating an unweighted graph by applying the maximum clique threshold (mct) to the weighted co-cluster graph such that a probe pair, or edge, was connected in the unweighted graph only if the corresponding weight in the co-cluster graph equaled or exceeded this threshold.

For the inner loop, the first step was to isolate the largest set of probes such that all probes in the set were completely connected in the unweighted graph. In graph theoretical terms, the probes form a maximum clique. An additional constraint was imposed that all probes in the set must co-cluster in the same datasets at least mct times by taking the intersection of the bitmasks for every edge in the clique and the result must contain at least mct bits set to 1. This new bitmask was the common co-cluster bitmask for the clique.

If the size of the clique was smaller than the minimum seed size, the inner loop was escaped, reduced mct by one, and the process returned to the beginning of the outer loop; otherwise, it became the seed for a module. To allow for inevitable clustering inaccuracies, the paraclique algorithm was used. The co-cluster graph was re-visited and added to the seed any probe that was found to co-cluster with at least 90% of the seed's members in at least pt of the datasets represented

in the clique's common co-cluster bitmask. This final probe set was a module: it was removed from both graphs and named in accordance with the iterations in which it was found (i.e. a module extracted in the first iteration of the outer loop and the second iteration of the inner loop is designated M1.2). The inner loop then began again with the reduced graphs. The Pseudocode is shown below:

```
Integer nLastQuartile = 4;
Integer nMaxRelaxtion = m_nNumDatasets / 3;
Integer nRelaxtionIncrement = Math.max(1, (nMaxRelaxtion / 3));
Integer nRelaxtion = nMaxRelaxtion;
for (int nCliqueThreshold = numberOfDatasets; nCliqueThreshold >= 1; nCliqueThreshold--)
{
	Integer nQuartile = ((nThreshold * 100) / m_nNumDatasets) / 25;
	if (nQuartile.equals(nLastQuartile) == false)
	{
		if (nQuartile <= 2)
		{
			nRelaxtion = Math.max(0, nRelaxtion - nRelaxtionIncrement);
		}
		nLastQuartile = nQuartile;
	}
	Integer nParacliqueThreshold = nThreshold - nRelaxtion;

	do
	{
		maximumClique = find maximum clique w co-clustering weight >= nCliqueThreshold
		if (size of maximumClique > 15)
		{
			paraclique = find paraclique in graph
			remove maximumClique and paraclique from graph
		}
	}   while (maximumClique is found)
```

Supplementary Tables

Supplementary Table 1: The characteristics of the input datasets used to construct three consecutive generations of blood transcriptome module repertoires.

	Generation 1	Generation 2	Generation 3
**Number of States**	8	7	16
**States**			
Systemic onset juvenile idiopathic arthritis	X	X	X
Systemic lupus erythematosus	X	X	X
Juvenile dermatomyositis			X
Type 1 Diabetes	X		
Multiple sclerosis			X
Kawasaki disease			X
COPD			X
Tuberculosis		X	X
Sepsis		X	X
Respiratory Syncytial Virus infection			X
Influenza virus infection	X		X
Human Immunodeficiency Virus infection		X	X
Escherichia coli infection	X		
Staphylococcus aureus infection	X		X
B-cell deficiency			X
Liver transplant recipients	X	X	X
Metastatic melanoma	X	X	X
Pregnancy			X
**Number of input datasets**	8	9	16
**Number of individual profiles**	239	410	985
**Sample source**	PBMCs	Whole Blood	Whole Blood
**Platform**	Affymetrix U133A&B	Illumina Hu6 v2	Illumina HT12 v3.0
**Rounds of module selection**	3	8	15
**Number of modules**	28	260	382
**Year published & reference**	2008 (18)	2013 (17)	Current work

Supplementary Table 2: Links to module aggregates annotation pages

Aggregate	Function	Links
**A1**	Lymphocytic	https://prezi.com/view/sxap39tKxkmCNTTNIlVO/
**A2**	TBD[#]	https://prezi.com/view/96GWajx5mZjuRS4B6gjA/
**A3**	TBD	https://prezi.com/view/OWFVI51FND0WWwNgsgJZ/
**A4**	TBD	https://prezi.com/view/2Zbq8ZDYbO4hbUd4r2KF/
**A5**	Lymphocytic	https://prezi.com/view/62tgA5E6roOlk5DRNvS1/
**A6**	Lymphocytic	https://prezi.com/view/Uks2Nd4lvizNNFVPtBEy/
**A7**	TBD	https://prezi.com/view/kKfergNj0SkLXyFtm0Dg/
**A8**	TBD	https://prezi.com/view/Y4uk1RPJyNcSndJYnFX6/
**A9**	TBD	https://prezi.com/view/jgYehQ9QhyADAttEsdoI/
**A15**	TBD	https://prezi.com/view/jgYehQ9QhyADAttEsdoI/
**A16**	TBD	https://prezi.com/view/SKzHeA0XYdLYvy2sY8gP/
**A17**	TBD	https://prezi.com/view/FS7sE1Vqew5g8EKOM1AM/
**A18**	TBD	https://prezi.com/view/aZMLflMNVrV7JnVaIILm/
**A24**	Oxidative phosphorylation	https://prezi.com/view/eiXvf2LNBLFRgrtaeCuM/
**A25**	TBD	https://prezi.com/view/pwyojaU62Z7GT102ZYwM/
**A26**	TBD	https://prezi.com/view/9CErpW3NwpN2HgRS3Hzf/
**A27**	Cell cycle	https://prezi.com/view/GgIiA0K9kSFHbpVj2I85/
**A28**	Interferon	https://prezi.com/view/E34MhxE5uKoZLWZ3KXjG/
**A29**	TBD	https://prezi.com/view/W4TShTd32dEJx0XPOF1U/
**A30**	TBD	https://prezi.com/view/kl7VHoJTWug0sn7TgXut/
**A31**	TBD	https://prezi.com/view/GqtUO22JJlSf16zMJKbB/
**A32**	TBD	https://prezi.com/view/qlbG9VFzegOndQKD8aiy/
**A33**	Inflammation	https://prezi.com/view/VBqKqHuLWCra3OJOIZRR/
**A34**	TBD	https://prezi.com/view/HcSgIEGP3TJjTSpaPCxv/
**A35**	Inflammation	https://prezi.com/view/7Q20FyW6Hrs5NjMaTUyW/
**A36**	Erythroid	https://prezi.com/view/M7dnztl2h61gXrKFQeB2/
**A37**	Erythroid	https://prezi.com/view/YyQs4WiXSNf0YXE79lfS/
**A38**	Erythroid	https://prezi.com/view/0KUlPICKUZGeUjb206R5/

[#] TBD = To be Determined

Supplementary Figures

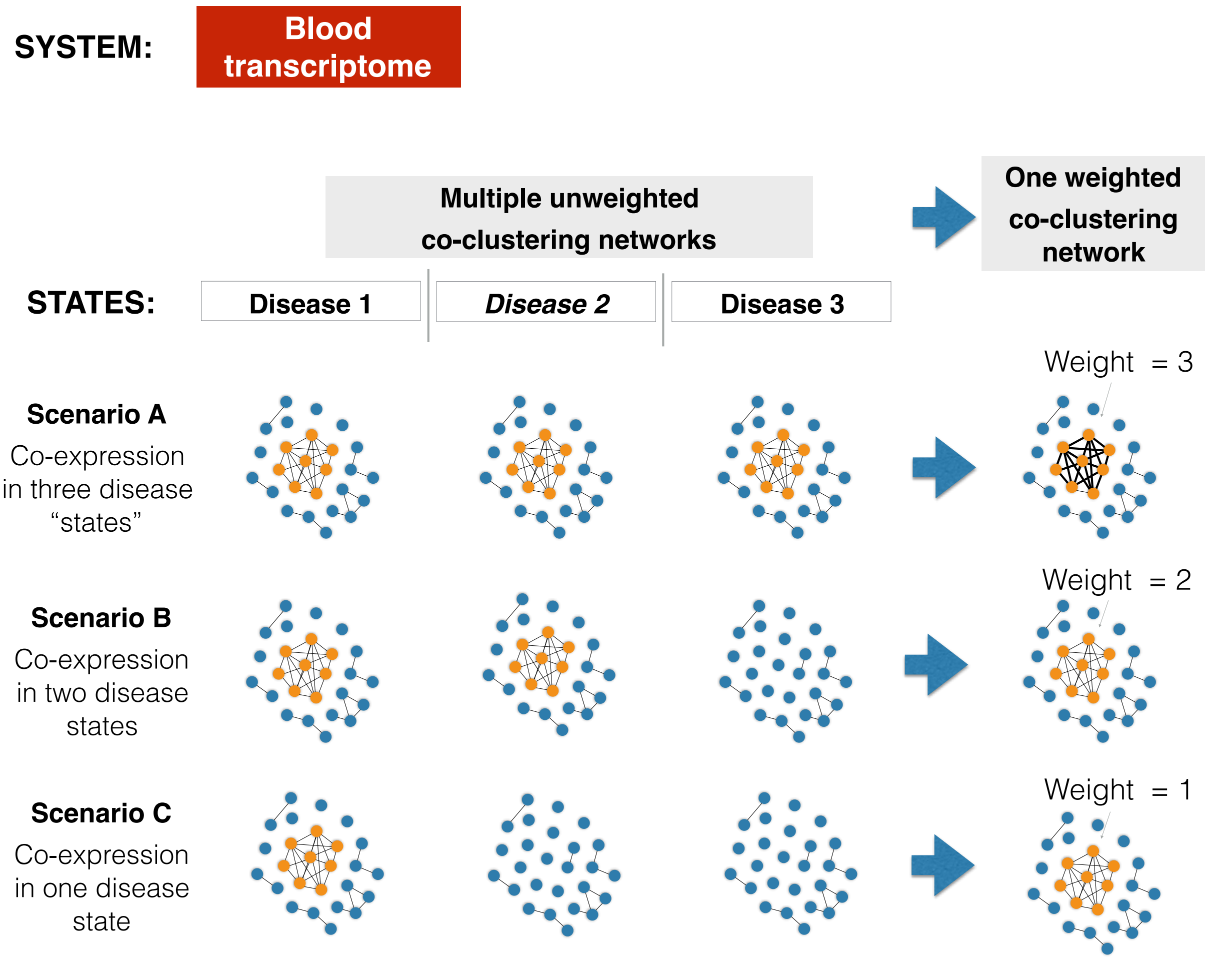

Supplementary Figure 1: Construction of weighted co-clustering networks. Weighted co-clustering networks were used to construct the BloodGen3 module repertoire. These networks factor in differences in co-expression across different "states" of the biological system. For the blood transcriptome, these states are different diseases or physiological phenotypes. Under Scenario A, the genes are co-expressed in all three disease states, so the weight attributed to the edges of the network is three. Under Scenarios B and C, co-clustering occurs in only two or one of the disease states, resulting in weights of 2 and 1, respectively. The image representing blood cells at the top

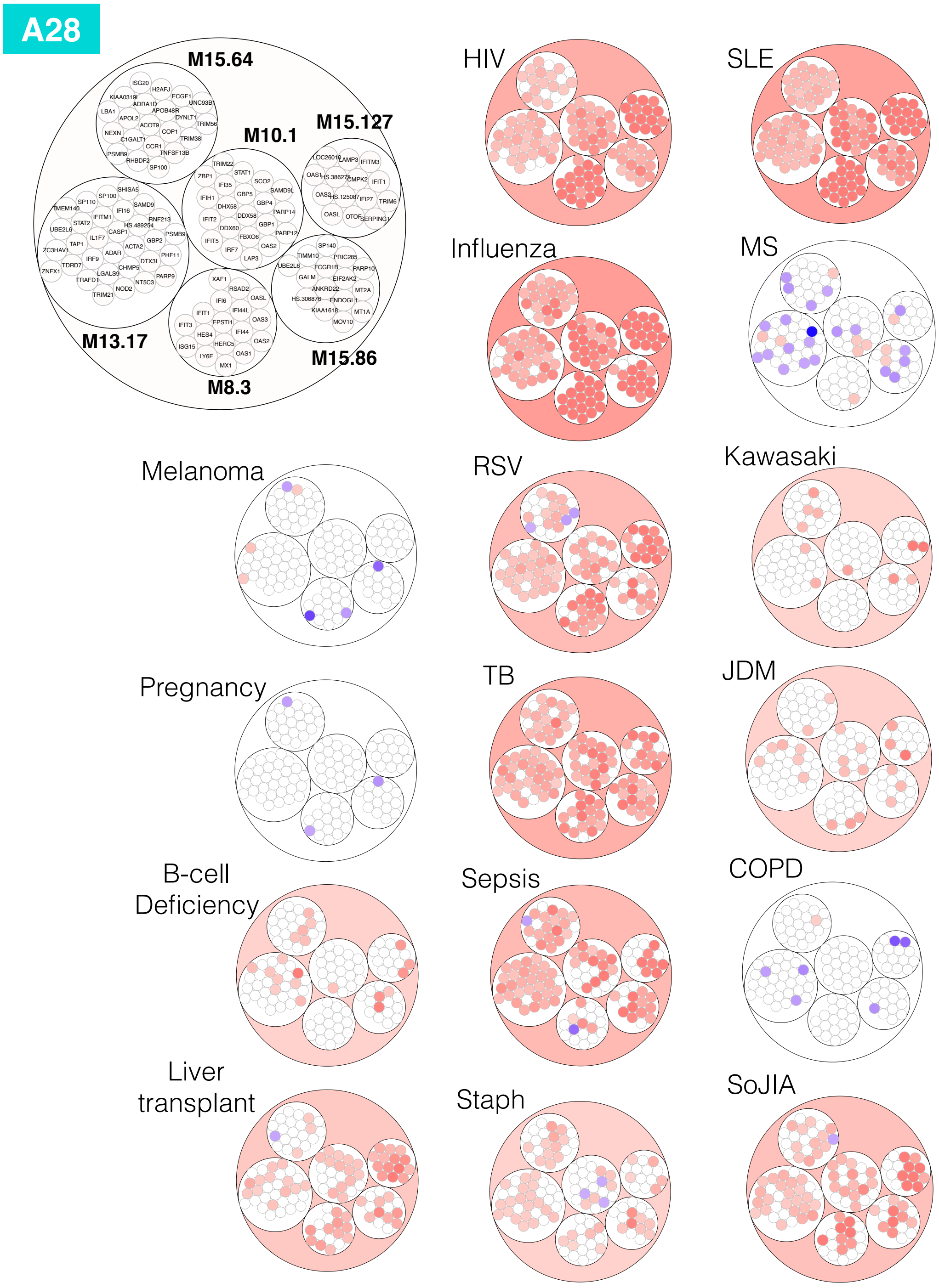

Supplementary Figure 2: Patterns of changes in transcript abundance at the module- and gene-levels for aggregate A28. The large circle plot represents the six modules constituting aggregate 28, and the transcripts constituting each of the modules. Some genes on the Illumina BeadArrays can map to multiple probes, which explains the few instances where the same gene can be found in different modules. Patterns of changes in transcript abundance at the module and gene-levels for aggregate A28 are shown for all 16 reference datasets. The position of the genes on each of these plots is fixed. Genes for which transcript abundance is changed are shown in red (increase) or in blue (decrease).

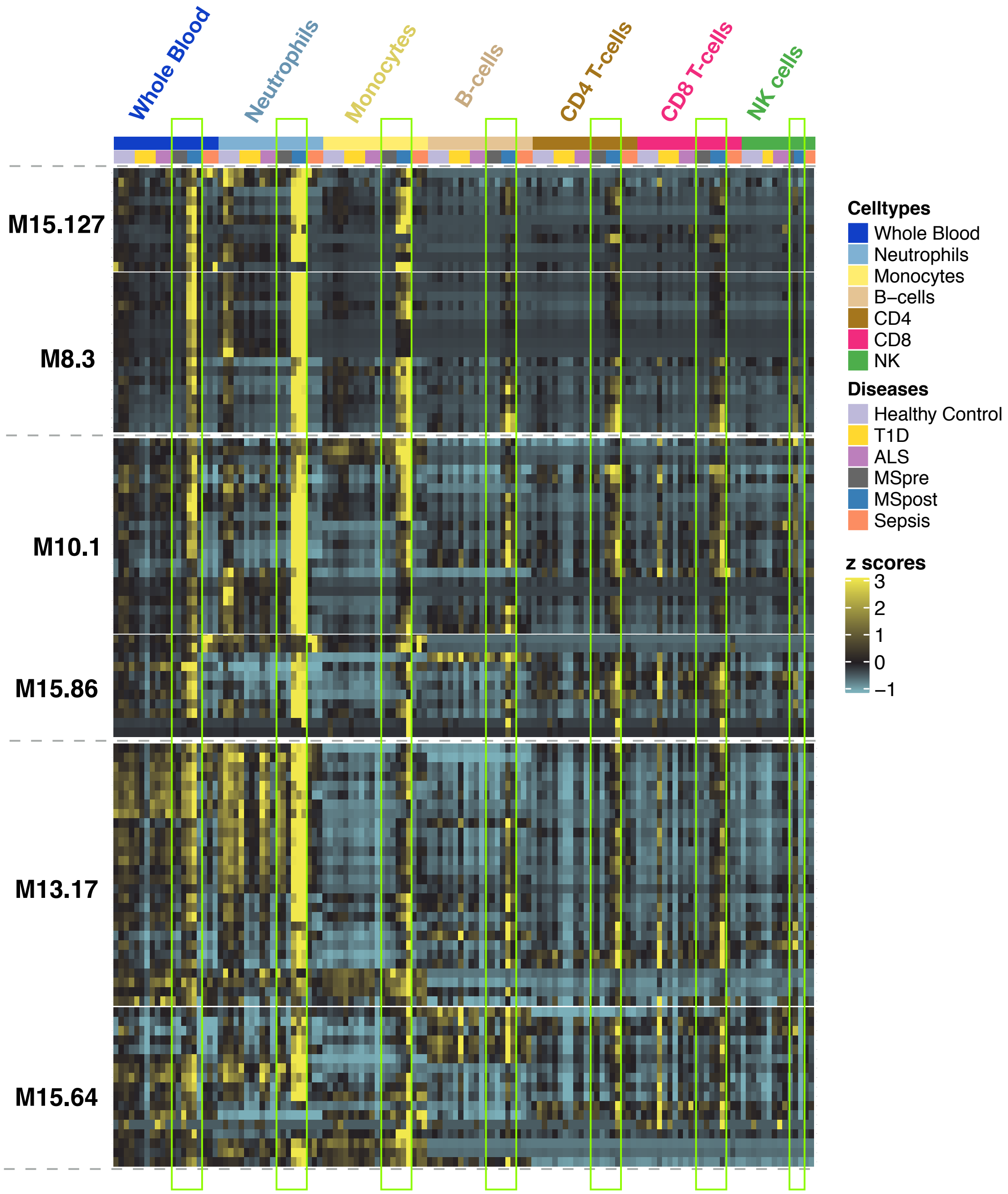

Supplementary Figure 3: Expression patterns of genes constituting the six A28 modules (rows) across whole blood and purified circulating leukocyte populations (columns). This dataset was contributed by our group [Speake *et al.* (GSE60424)], and compared the RNA-seq transcriptome signatures of six immune-cell subsets and whole blood from patients with various immune-associated diseases (color-coding is provided to indicate cell populations and health status). Aggregate A28 is comprised for the most part of well characterized interferon-inducible modules. The columns highlighted by the green boxes show the expression levels in patients with MS before and 24 h after the first dose of IFN-β.

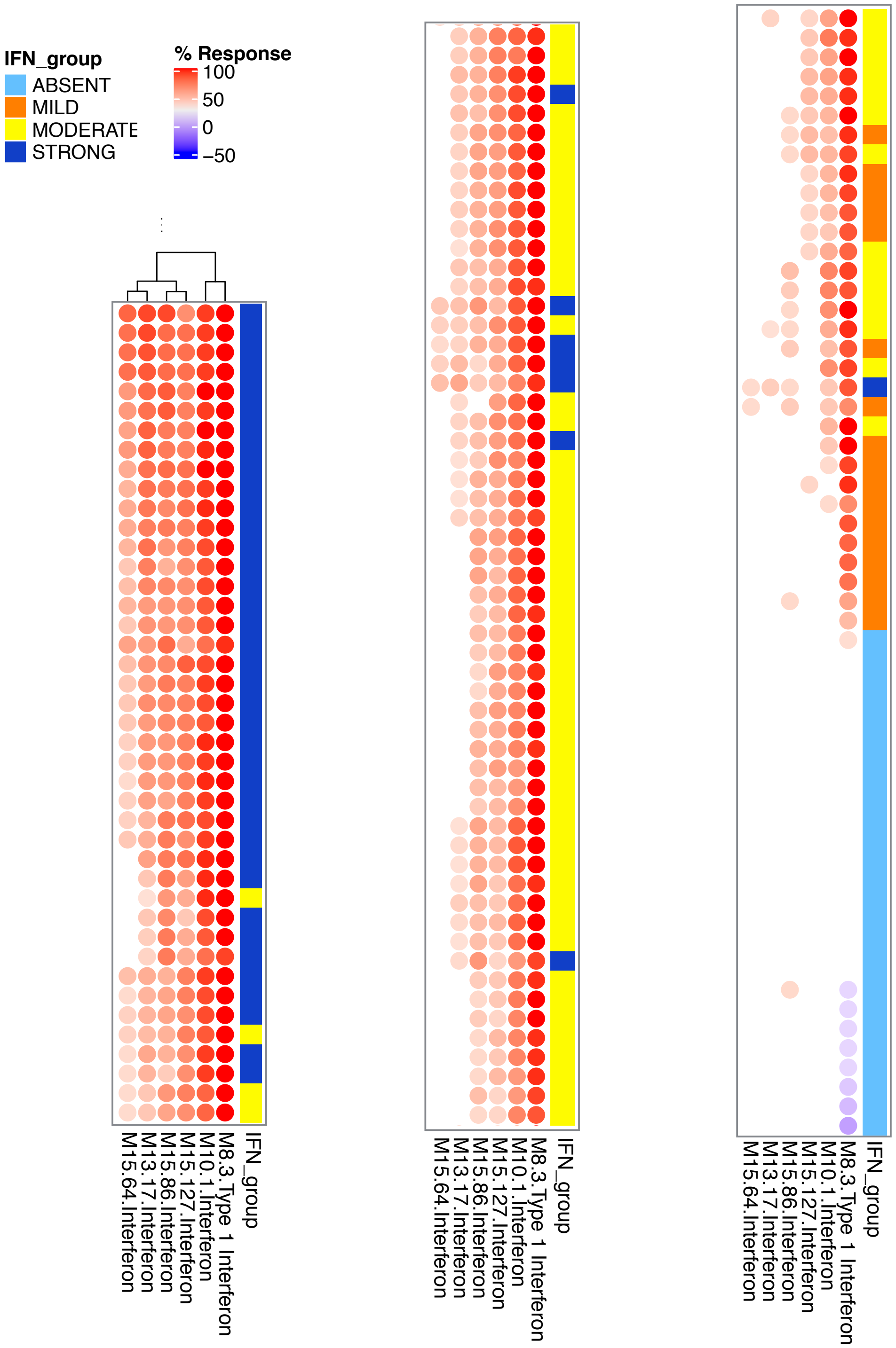

Supplementary Figure 4: Transcript abundance patterns of six interferon modules (A28) across individuals from an adult SLE cohort. The transcript abundance levels for six interferon modules belonging to aggregate A28 (columns) is shown across a cohort of adult subjects diagnosed with SLE (rows). These subjects were part of the LUPUCE study: the dataset was contributed by our group [Chiche *et al.* (GEO ID GSE49454)]. Changes in transcript abundance were measured in reference to a cohort of healthy subjects included in the study. The colored label with interferon grouping information indicates the classification obtained by Chiche *et al.* based on three “second-generation” modules.
