# Supplementary material for: Development of a fixed module repertoire for the analysis and interpretation of blood transcriptome data
Source: Nat Commun. 2021 Jul 19;12:4385. doi: 10.1038/s41467-021-24584-w (PMC8289976; doi:10.1038/s41467-021-24584-w)
Supplement: Supplementary file 3 — Description of Additional Supplementary Files [file 41467_2021_24584_MOESM3_ESM.pdf]

Description of Additional Supplementary Files

File Name: Supplementary Data 1

Description: Annotated module repertoire

This Excel spreadsheet lists the 382 modules constituting this third generation of the blood transcriptome module repertoire. The number of genes, the list of member genes by symbol and the probe ID, and summarized functional annotations are included. Module functional annotations will be updated over time, as our understanding of the biological underpinnings of this repertoire grows. The most up-to-date module annotation will be found on our GitHub project page (https://rdrr.io/github/Drinchai/BloodGen3Module/) and changes will be reflected in the Prezi annotation pages. However, the module composition and positioning on the grid will remain unchanged.
