# Supplementary material for: Development of a fixed module repertoire for the analysis and interpretation of blood transcriptome data
Source: Nat Commun. 2021 Jul 19;12:4385. doi: 10.1038/s41467-021-24584-w (PMC8289976; doi:10.1038/s41467-021-24584-w)
Supplement: Supplementary file 5 — Reporting Summary [file 41467_2021_24584_MOESM5_ESM.pdf]

nature research

Corresponding author(s): Damien Chaussabel

Last updated by author(s): Jun 2, 2021

Reporting Summary

Nature Research wishes to improve the reproducibility of the work that we publish. This form provides structure for consistency and transparency in reporting. For further information on Nature Research policies, see our Editorial Policies and the Editorial Policy Checklist.

Statistics

For all statistical analyses, confirm that the following items are present in the figure legend, table legend, main text, or Methods section.

n/a	Confirmed	
☐	☒	The exact sample size (*n*) for each experimental group/condition, given as a discrete number and unit of measurement
☒	☐	A statement on whether measurements were taken from distinct samples or whether the same sample was measured repeatedly
☐	☒	The statistical test(s) used AND whether they are one- or two-sided<br>*Only common tests should be described solely by name; describe more complex techniques in the Methods section.*
☒	☐	A description of all covariates tested
☐	☒	A description of any assumptions or corrections, such as tests of normality and adjustment for multiple comparisons
☐	☒	A full description of the statistical parameters including central tendency (e.g. means) or other basic estimates (e.g. regression coefficient) AND variation (e.g. standard deviation) or associated estimates of uncertainty (e.g. confidence intervals)
☒	☐	For null hypothesis testing, the test statistic (e.g. *F*, *t*, *r*) with confidence intervals, effect sizes, degrees of freedom and *P* value noted<br>*Give P values as exact values whenever suitable.*
☒	☐	For Bayesian analysis, information on the choice of priors and Markov chain Monte Carlo settings
☒	☐	For hierarchical and complex designs, identification of the appropriate level for tests and full reporting of outcomes
☒	☐	Estimates of effect sizes (e.g. Cohen's *d*, Pearson's *r*), indicating how they were calculated

Our web collection on statistics for biologists contains articles on many of the points above.

Software and code

Policy information about availability of computer code

Data collection	No Software was used for data collection.
Data analysis	A R package was developed that supports module repertoire analyses and fingerprint visualizations described in the manuscript. It is available on GitHub [1] and Bioconductor [2] and described in more details in a separate publication [3]<br>[1] https://github.com/Drinchai/BloodGen3Module<br>[2] https://bioconductor.org/packages/release/bioc/html/BloodGen3Module.html<br>[3] https://pubmed.ncbi.nlm.nih.gov/33624743/

For manuscripts utilizing custom algorithms or software that are central to the research but not yet described in published literature, software must be made available to editors and reviewers. We strongly encourage code deposition in a community repository (e.g. GitHub). See the Nature Research guidelines for submitting code & software for further information.

Data

Policy information about availability of data

All manuscripts must include a data availability statement. This statement should provide the following information, where applicable:

- Accession codes, unique identifiers, or web links for publicly available datasets
- A list of figures that have associated raw data
- A description of any restrictions on data availability

Transcriptome profiling data that support the findings of this study have been deposited in the NCBI Gene Expression Omnibus (GEO) with the accession code GSE100150 (https://www.ncbi.nlm.nih.gov/geo/query/acc.cgi?acc=GSE100150).

Field-specific reporting

Please select the one below that is the best fit for your research. If you are not sure, read the appropriate sections before making your selection.

☒ Life sciences ☐ Behavioural & social sciences ☐ Ecological, evolutionary & environmental sciences

For a reference copy of the document with all sections, see nature.com/documents/nr-reporting-summary-flat.pdf

Life sciences study design

All studies must disclose on these points even when the disclosure is negative.

Sample size	The main objective of this work was the development of a repertoire of transcriptional modules. This is based on co-expression and as such does not rely on group comparison or statistical hypothesis testing. We instead constructed a weighted co-expression networks using k-means clustering. It was subsequently mined using graph theory algorithms. The minimum number of cases for a given dataset subjected to clustering was empirically set to 20 based on two earlier repertoires built by our group and QCing of clustering performed for different sample sizes [Chaussabel et al. Immunity. 2008 Jul 18;29(1):150-64], [Obermoser et al. Immunity 2013 Apr 18;38(4):831-44].
Data exclusions	Pre-established filtering criteria were applied to remove transcripts that either show minimal detection levels or minimal changes in expression across subjects (criteria employed are detailed in the methods section); briefly: "First, the probes were discarded if they were not present (detection P < 0.01) in at least 10 samples or in at least 10% of the samples, whichever was greater. " & "Probes were only retained if they had a calculated absolute fold change >1 in at least 10 samples or in at least 10% of the samples, whichever was greater. "
Replication	The co-expression network that we have built is based on co-clustering patterns observed across 16 independent datasets, each comprising at least 20 subjects (biological replicates). For any gene-pair a weigh was added to the network to indicate the number of instance where co-clustering was observed (ranging in value between 16 = co-clustering observed in all datasets to 1 = co-clustering observed in one dataset). The module construction process takes into account the degree of robustness of clustering since the gene set selection starts with subnetworks presenting the highest weight (16 out of 16), while the stringency is being progressively relaxed at each round of selection (from modules denoted as M1 = first picks, highest weight, to M16 last pick, lowest weight)
Randomization	Randomization did not apply here. The work is based on a series of observational case-control studies. Subjects were assigned to a group (case or control) as a function on their health status. The studies did not have treatment/intervention arms to which subjects would be assigned to.
Blinding	The studies were observational and did not include treatment/interventions investigators would be blinded for.

Reporting for specific materials, systems and methods

We require information from authors about some types of materials, experimental systems and methods used in many studies. Here, indicate whether each material, system or method listed is relevant to your study. If you are not sure if a list item applies to your research, read the appropriate section before selecting a response.

Materials & experimental systems

n/a	Involved in the study	
☒	☐	Antibodies
☒	☐	Eukaryotic cell lines
☒	☐	Palaeontology and archaeology
☒	☐	Animals and other organisms
☐	☒	Human research participants
☒	☐	Clinical data
☒	☐	Dual use research of concern

Methods

n/a	Involved in the study	
☒	☐	ChIP-seq
☒	☐	Flow cytometry
☒	☐	MRI-based neuroimaging

Human research participants

Policy information about studies involving human research participants

Population characteristics	In this study transcriptome profiles were generated from 16 reference cohorts of patients. Covariate-relevant population characteristics of human research participants included age, gender and ethnicity.
Recruitment	Written informed consent was obtained from all participants. Given the range of studies involved it would be best to refer to the method section of the manuscript for details about criteria specific to each study. Groups of control subjects were enrolled to account for recruitment biases that might inherently impart each cohort with distinct demographic characteristics (e.g. gender in the case of the pregnancy cohort or ethnicity in the case of the sepsis cohort).

Ethics oversight	Studies were approved by Institutional Review Boards of the Baylor College of Medicine (COPD dataset: H-18029), the University of Texas Southwestern Medical Center and Baylor Health Care System (Influenza, RSV, S. aureus and Kawasaki disease datasets: UTSW #0802-447 / BIIR #002-141), Saint Jude's Research Hospital (B-cell deficiency), the Baylor Health Care System (Liver transplant: 002-197, Pregnancy: 009-257, Multiple sclerosis: 009-240, Melanoma: 006-025 & 097-027), Khon Kaen University (Sepsis), the University of Texas Southwestern Medical Center (SoJIA, Dermatomyositis, SLE), Duke University and the Baylor Health Care System (HIV: Duke 8485-06-4R0 / Baylor 006-177), St. Mary's Hospital London, UK and University of Cape Town, Cape Town, Republic of South Africa (Tuberculosis: St Mary's REC 06/Q0403/128, U of Cape Town REC 012/2007).

Note that full information on the approval of the study protocol must also be provided in the manuscript.
